# Protocol of a study investigating breath-hold techniques for upper-abdominal radiation therapy (BURDIE): addressing the challenge of a moving target

**DOI:** 10.1186/s13014-020-01688-z

**Published:** 2020-10-30

**Authors:** Briana Farrugia, Richard Khor, Farshad Foroudi, Michael Chao, Kellie Knight, Caroline Wright

**Affiliations:** 1 Radiation Oncology, Olivia Newton-John Cancer Wellness and Research Centre, Austin Health, PO Box 5555, Heidelberg, VIC 3084 Australia; 2 Department of Medical Imaging and Radiation Sciences, Faculty of Medicine, Nursing and Health Sciences, Monash University, Wellington Rd, Clayton, VIC 3800 Australia

**Keywords:** Breath holding, Neoplasms, Radiotherapy

## Abstract

**Background:**

Radiation therapy to upper abdominal sites is technically challenging due to motion of tumors and surrounding organs resulting from normal respiration. Breath-hold, using an Active Breathing Coordinator is one strategy used to reduce motion in these tumor sites. Though widely used, no studies have prospectively compared the different breath-hold techniques (inspiration, deep-inspiration and expiration) using ABC in the same patient cohort.

**Methods:**

Patients planned for radiation therapy to upper abdominal tumors are invited to participate in this prospective study. Participants attempt three breath hold techniques: inspiration, deep-inspiration and expiration breath-hold, in random order. kV fluoroscopy images of the dome of diaphragm are taken of five consecutive breath-holds in each technique. Reproducibility and stability of tumour position are measured, and used to select the technique with which to proceed to planning and treatment. Reproducibility at planning and each treatment fraction is measured, along with breath hold time, treatment efficiency and patient experience.

**Discussion:**

The screening method was validated after the first three participants. This screening process may be able to select the best breath-hold technique for an individual, which may lead to improved reproducibility. The screening process is being piloted as a prospective clinical trial.

***Trial registration*:**

Australian New Zealand Clinical Trials Registry (ANZCTR): 12618001691235. Registered 12th October 2018. https://www.anzctr.org.au/Trial/Registration/TrialReview.aspx?id=376109&isReview=true.

## Background

Radiation therapy (RT) to upper abdominal (UA) sites, including liver, pancreas, kidneys and adrenal glands, is technically challenging. This is due to the proximity of the tumor to organs at risk (OAR), and OAR motion due to both respiration and physiological variation, such as filling of gastro-intestinal organs [1].

Breath-hold (BH) techniques, either voluntary or assisted, have been implemented to minimize respiratory-induced motion [2–7]. Inspiration Breath-Hold (IBH), Deep-Inspiration Breath-Hold (DIBH) and Expiration Breath-Hold (EBH) are reported in the literature [3–7]. Employing a voluntary IBH technique has demonstrated cohort reproducibility (R_BH_) of 4–10 mm [3, 8, 9], whilst voluntary EBH has demonstrated cohort R_BH_ of 2–5 mm [3, 9]. When an Active Breathing Coordinator (ABC)™ device (Elekta, Stockholm, Sweden) is used to assist breath-hold, improvements in R_BH_ have been seen, with DIBH intra-fraction cohort R_BH_ of 1.3–1.6 mm [6, 7], and EBH intra-fraction cohort R_BH_ of 1.5 mm [10]. Although EBH techniques tend to display a better R_BH_ at a cohort level, in many studies the R_BH_ ranges for different BH techniques overlap [3, 9]. This suggests that patients may be able to perform multiple breath hold techniques adequately enough for IGRT. Although population level estimates for the average cohort R_BH_ and S_BH_ have been done, none yet aim to select the best method for each patient.

There is limited literature available to describe patients’ experience of BH. A recent study evaluated 150 patients’ experiences of voluntary BH using MR-guided RT using an un-validated questionnaire [11]. Considerable difficulty controlling their tumor position in voluntary BH was reported by 12.5% of patients [11]. Another study investigated the patient experience of DIBH, in 41 patients receiving breast RT [12]. More than 90% of participants rated their experience > 8 on a 10 point Likert-type scale (where 0 = not at all; 10 = extremely) for ease, comfort and control of BH using ABC [12]. To our knowledge, no studies have investigated patients’ experiences of multiple BH techniques.

## Methods

### Study design and ethics

**Fig. 1:**
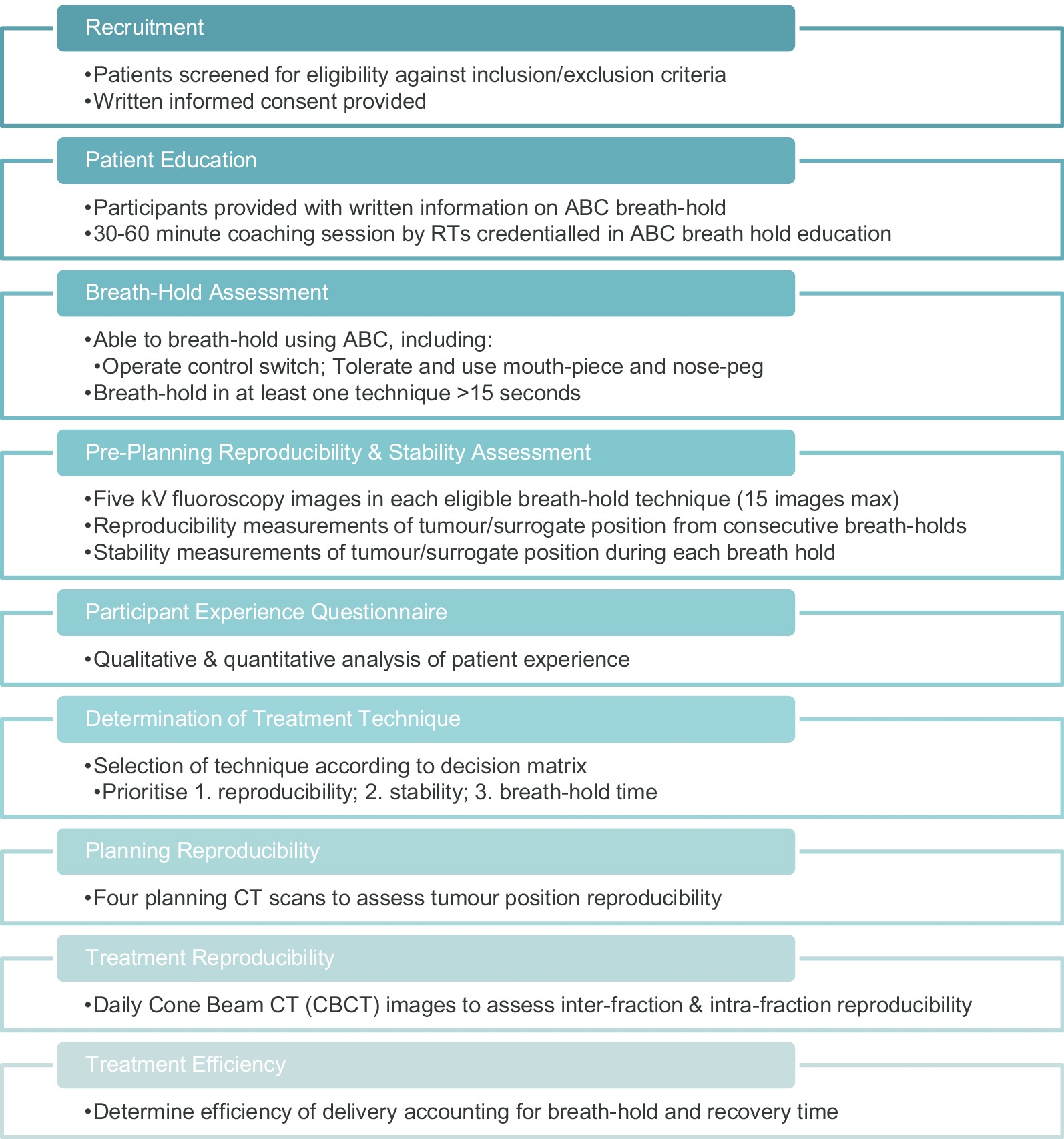
Study schema

The aims of this study are to evaluate the R_BH_ and stability (S_BH_) of tumor position; patient experience; and efficiency of treatment delivery for each BH technique. This is a prospective, single-institution study of adults, aged over 18, undergoing RT for malignancies of the liver, pancreas, adrenal gland and kidney. Patients suitable for RT, including Stereotactic Ablative Radiotherapy (SABR) techniques are eligible and invited to participate. The study was approved by our institutional Human Research Ethics Committee (HREC 47012). Once identified, eligible participants are provided with written information about the study from their Radiation Oncologist (RO) or the study coordinator. Informed consent is mandated prior to enrolment. Figure 1 provides an overview of study procedures.

### Primary outcome measures

a R_BH_ and S_BH_ of UA tumors in IBH, DIBH and EBH, measured pre-planning.
b Number of participants screened into each BH technique.
c R_BH_ of UA tumors at planning, in participant’s selected technique.
d Inter-fraction and intra-fraction R_BH_ of UA tumors at each treatment fraction, in participant’s selected technique.

### Secondary outcome measures

a Treatment efficiency of IBH, DIBH and EBH, in participant’s selected technique.
b Patient-reported experience of IBH, DIBH and EBH, measured pre-planning.

## Study procedure*s*

### Breath-Hold Assessment

**Fig. 2:**
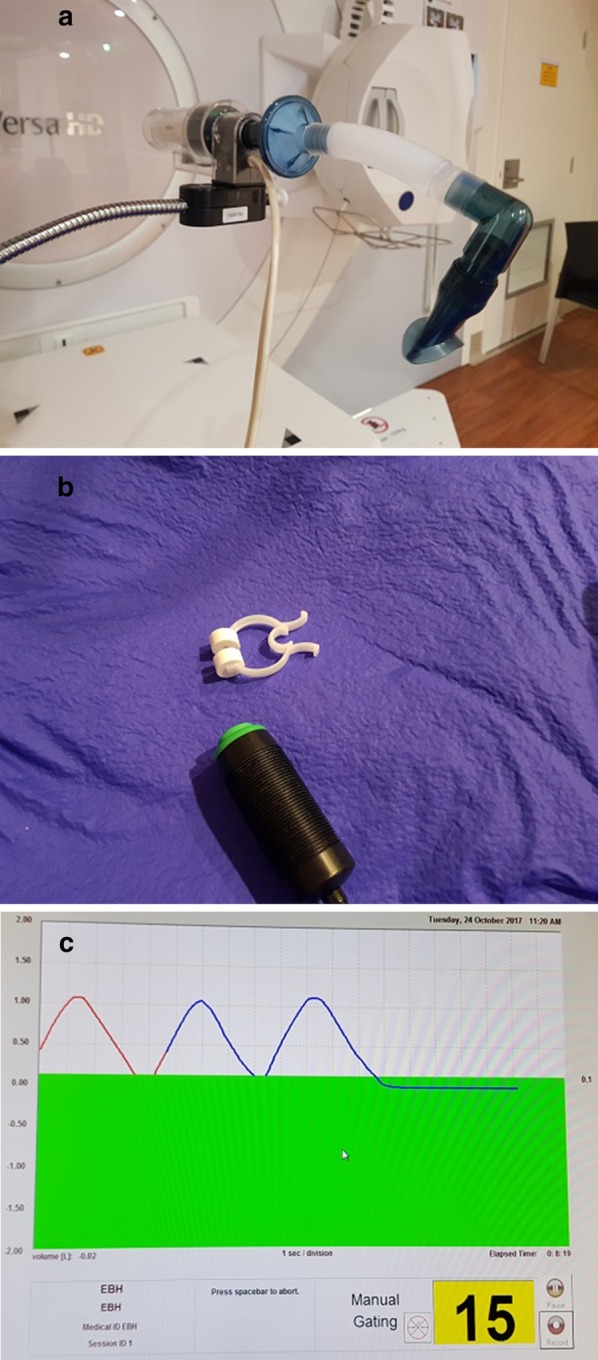
Active Breathing Coordinator™ components. **a** Mouth-piece and spirometer; **b** nose-peg and control switch; **c** operators screen displaying activated EBH indicated by green bar

All participants undergo protocolled education with ABC, then an assessment to confirm their ability to ABC-BH (Fig. 2a–c: ABC Device). Participants are screened for eligibility in IBH, DIBH and EBH in random order to reduce risk of bias, using a pre-determined block randomization sequence. Eligibility, breath-hold time (T_BH_) and ABC-BH threshold are recorded for each technique.

*T*_*BH*_ Time (seconds) that the participant can BH comfortably, at least three times.

*ABC-BH Threshold* Volume of air (liters) in the participant’s lungs at BH activation. The radiation therapists determine the threshold with the participant, as follows:

- *EBH* 0.0–0.2 L
- *IBH* Peak volume of air during normal, relaxed respiration. Average of three measurements.
- *DIBH* Maximum peak volume of air during voluntary deep inspiration. Threshold is 80% of average of three measurements.

### Pre-planning reproducibility and stability

**Fig. 3:**
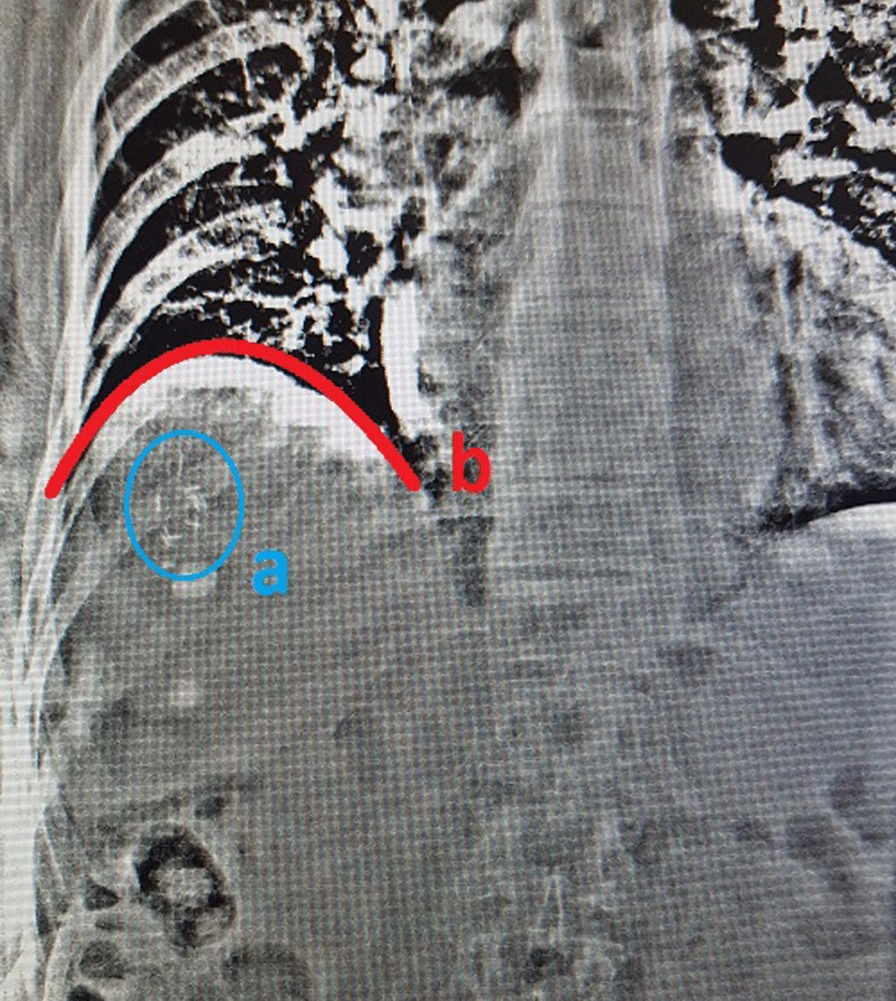
AP kV fluoroscopy image with fiducial markers (**a**—blue) and diaphragm position (**b**—red)

To assess R_BH_ of tumor position between consecutive BHs, and S_BH_ of tumor position during each BH, 5 kV X-ray fluoroscopy images are acquired in each BH technique on an Elekta™ linear accelerator, using the XVI™ software “MotionView” function. The position of the tumor, or an appropriate surrogate (diaphragm or fiducial marker), is tracked. Each image is acquired anterior–posteriorly (AP) (Fig. 3), for the complete duration of each of the participant’s BH’s. R_BH_ and S_BH_ are defined as follows:

- *R*_*BH*_ Cranio-caudal position of the tumor/surrogate at the beginning of BH1 is compared to the position at beginning of BH2-5. Four observations are recorded per technique. The absolute value (ignoring direction) of the cumulative sum of each observation is determined, and then averaged.
- *S*_*BH*_ Cranio-caudal displacement of the tumor/surrogate, during each BH. Five observations are recorded per technique. The mean of the absolute value (ignoring direction) of each observation is then calculated.

### Patient experience questionnaire

Participants are invited to complete a questionnaire (Additional file 1: Appendix 1) to evaluate their experience of each technique. The questionnaire, developed specifically for this study, as there was no available validated questionnaire, includes both quantitative questions using a Likert-type scale; and qualitative open-ended questions, allowing the participant to elaborate on their experience. Participants are asked to rank the techniques in order of their preference. As required, a staff member involved in the breathing and/or reproducibility and stability assessment will conduct a semi-structured interview with the patient to elicit responses to all applicable questions.

### Determination of treatment technique

**Fig. 4:**
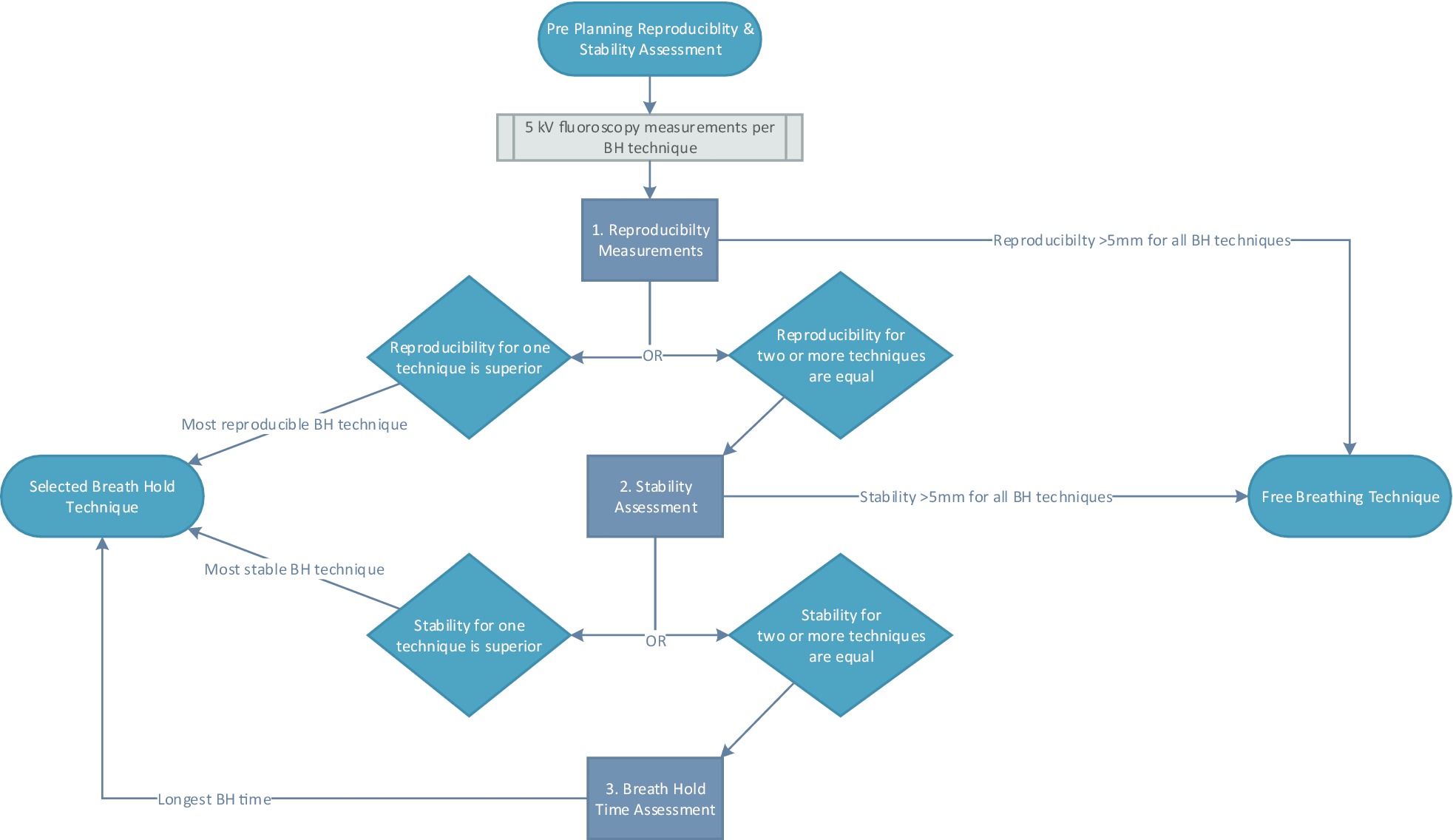
Decision matrix for selection of treatment technique

Following the ABC-BH and pre-planning R_BH_ and S_BH_ assessments, selection of treatment technique is made according to the decision matrix (Fig. 4), with R_BH_ prioritized. If two or more techniques have equal (to nearest mm) mean R_BH_, then the technique with better S_BH_ is selected. A technique with mean R_BH_ and/or S_BH_ > 5 mm will proceed to free-breathing.

### Planning reproducibility

Participants have all subsequent planning and treatment using the chosen BH technique. Four planning computed tomography (CT) scans are acquired for each participant. The scans are acquired sequentially within a 5 min timeframe, with no patient-repositioning between scans, to minimize the impact of repositioning or physiological variation. The Gross Tumor Volume (GTV), tumor surrogates and OARs are contoured on the first planning CT. Post-processing and measurements are completed using a customized workflow in MIM Maestro® software (MIM Software Inc, Cleveland OH). The first planning CT is automatically fused to subsequent CTs. Initially, a bone algorithm fusion corrects any gross patient misalignment. Then, a tumor, surrogate or fiducial marker fusion corrects for tumor/organ position. Contours are automatically generated onto each subsequent CT. Resultant contours are reviewed, and manually edited if required to account for deformation. The inter-BH displacement of the tumor and OARs are measured, with bone fusion as the starting position. Planning R_BH_ of tumour/surrogate and OARs is defined as follows:

- *R*_*BH*_ Position, in three planes, of the tumor/surrogate or OAR contour centroid and contour surface in CT1 compared to the position in CT2-4, resulting in three observations. The mean of the absolute values of the cumulative sum of each observation will be calculated.

### Treatment reproducibility

Intra-fraction and inter-fraction R_BH_ is measured for each participant. For all treatment fractions, 3D volumetric Cone Beam Computed Tomography (CBCT) images are acquired using Elekta XVI™ (Elekta, Stockholm, Sweden) before treatment delivery. For participants treated with SABR, post-correction, during, and post-treatment CBCTs are also acquired. Each CBCT is a 360° acquisition using several BHs, with acquisition paused between BHs. The CBCT is automatically fused to the planning CT with a bone algorithm to correct patient setup. Then, a soft-tissue fusion corrects for tumor position. Tumor displacement is measured as the correction applied after accounting for patient setup. Treatment R_BH_ of tumour is defined as follows:

- *Inter-Fraction R*_*BH*_ Position, in three planes, of the tumor/surrogate on planning CT compared to the position on CBCT1, at each treatment fraction. Results will be collated for all fractions, and averaged.
- *Intra-Fraction R*_*BH*_ For those participants with multiple CBCTs per fraction, the position, in three planes, of the tumor/surrogate on CBCT1 compared to the position on each subsequent CBCT, at each treatment fraction. The mean values of the absolute value of the cumulative sum of each observation will be calculated.

### Treatment efficiency

Total treatment time, T_BH_, and number of BHs required to deliver treatment are recorded for the first three fractions for all participants. From this, an estimate of treatment efficiency is determined, as the proportion of total treatment time in which delivery occurred.

## Statistical analysis

Sample size calculation indicates required recruitment of 14–27 participants to be powered to detect a 2 mm variation in reproducibility, assuming standard deviation of 2 mm or 3 mm respectively.

The R_BH_ and S_BH_ measurements during pre-planning will be collated and described for each participant and each BH method. The number of patients screened into each BH technique will be reported, along with descriptive statistics such as the mean and standard deviation (SD) for R_BH_ and S_BH_ in order to compare each technique. Paired *t* tests will be used to test for differences between the BURDIE-screened R_BH_ and S_BH_ with our institutional standard technique EBH, including subset analysis of those participants who do not screen into EBH. Further comparisons will be made to compare each BH technique’s mean R_BH_ and S_BH_ with the selected technique mean. Mixed effects models will be used to evaluate the multiple observations for each patient, and each technique.

The planning mean R_BH_ measurements will be compared with the pre-planning results for each participant, and allow an assessment of the correlation between the mean R_BH_ values at the two time-points. Paired *t* tests will be conducted to provide an estimate of the 95% confidence interval in the paired observations with a margin of ± 2 mm considered to reflect similarity and/or an indication of the non-inferiority margin. Bland–Altman plots, with Pitman’s test will be prepared to test for any indication of bias in the pre-planning and planning R_BH_ means. Tests for variation across each BH technique will be conducted, with assessments of the average R_BH_ values for each technique, the study population and comparisons to the pre-planning time-point.

To evaluate treatment R_BH_, correlation coefficients will be estimated as fixed-effects, to indicate the consistency between the inter-fraction and intra-fraction observations. The treatment mean R_BH_, will be compared to the pre-planning and planning results, and allow an assessment of the correlation between these paired means. The 95% confidence interval in the paired observations will be compared to a non-inferiority margin of ± 2 mm, with Bland–Altman plots and the Pitman’s test used to test for any indication of bias in the treatment R_BH_ for comparison with pre-planning and planning means. However, it is expected that that the difference between the pre-planning, planning and treatment values may reduce over time, and these differences will be assessed descriptively, and compared to the non-inferiority margin of ± 2 mm.

### Feasibility assessment

The methodology was validated after the first three recruited participants. All three participants who were eligible and approached for the trial agreed to participate. All three were able to complete the Breath-Hold Assessment and Pre-Planning Reproducibility and Stability Assessment. A summary of these results is presented in Table 1, with IBH being the BURDIE-selected technique for all three participants.

**Table 1:** Results of feasibility assessment (n = 3)

Measure		Breath-hold technique mean (SD)		
		DIBH	IBH	EBH
*Participant 1*				
Tumour site	Kidney			
Age (years)	32			
Gender (M/F)	M			
ABC threshold (L)		1.4	0.6	0.1
T_BH_ (s)		16	16	16
S_BH_ (mm)		0.4 (0.5)	0.8 (0.4)	2.0 (1.6)
R_BH_ (mm)		4.0 (2.6)	0.5 (0.6)	1.5 (1.3)
Test order	EBH, IBH, DIBH			
Selected technique	IBH			
*Participant 2*				
Tumour site	Liver			
Age (years)	71			
Gender (M/F)	M			
ABC threshold (L)		1.2	0.9	0.1
T_BH_ (s)		35	35	30
S_BH_ (mm)		2.2 (1.5)	1.4 (0.5)	2.0 (2.3)
R_BH_ (mm)		1.0 (0.0)	0.25 (0.5)	0.75 (1.0)
Test order	DIBH, IBH, EBH			
Selected technique	IBH			
*Participant 3*				
Tumour site	Liver			
Age (years)	68			
Gender	M			
ABC threshold (L)		0.8	0.6	0.1
T_BH_ (s)		20	20	15
S_BH_ (mm)		0.6 (0.9)	0.6 (0.9)	0.8 (1.3)
R_BH_ (mm)		2.0 (2.3)	1.0 (0.8)	3.75 (1.0)
Test order	IBH, EBH, DIBH			
Selected technique	IBH			

*DIBH* deep-inspiration breath-hold, *IBH* inspiration breath-hold, *EBH* expiration breath-hold, *T*_*BH*_ time (duration) of breath-hold, *R*_*BH*_ reproducibility of breath-hold, *S*_*BH*_ stability of breath-hold

## Discussion

A process to compare and select the optimal BH technique for each individual patient, using ABC, was developed. This screening process may be able to select the best BH technique for an individual, which may lead to improved R_BH_. The screening process is being piloted as a prospective clinical trial. The results of this study will be disseminated through publication in peer-reviewed journal(s) and/or conference presentations.

## Supplementary information

**Additional file 1: Appendix 1**. Patient experience questionnaire.

## Data Availability

Not applicable.

## Abbreviations

ABC: Active Breathing Coordinator
BH: Breath-hold
CBCT: Cone-beam computed tomography
CT: Computed tomography
DIBH: Deep-inspiration breath-hold
EBH: Expiration breath-hold
GTV: Gross tumour volume
IBH: Inspiration breath-hold
kV: Kilovoltage
OAR: Organ at risk
RT: Radiation therapy
R_BH_: Reproducibility of breath-hold
RO: Radiation oncologist
SABR: Stereotactic ablative radiotherapy
S_BH_: Stability of breath-hold
T_BH_: Time of breath-hold
UA: Upper abdominal
