# Supplementary material for: Protocol of a study investigating breath-hold techniques for upper-abdominal radiation therapy (BURDIE): addressing the challenge of a moving target
Source: Radiat Oncol. 2020 Oct 30;15:250. doi: 10.1186/s13014-020-01688-z (PMC7602358; doi:10.1186/s13014-020-01688-z)
Supplement: Supplementary file 1 — Additional file 1: Appendix 1. Patient experience questionnaire. [file 13014_2020_1688_MOESM1_ESM.docx]

# **Participant Experience Questionnaire**

Date Completed: ____________

Order of tests (if applicable): 1. _____________ 2. _____________ 3. ______________

Staff initials: _______________

(Please provide questions for breath-hold method(s) attempted, in order of tests performed)

Please circle a number to indicate your response.

The following questions relate to your overall experience of **breath-hold**:

|  | **Not at all** | **Slightly** | **Neutral** | **Moderately** | **Very** | **N/A** |
| --- | --- | --- | --- | --- | --- | --- |
| 1. Were the spoken instructions easy to understand? | 1 | 2 | 3 | 4 | 5 |  |
| 2. Did the visual aid assist you to understand the spoken instructions? | 1 | 2 | 3 | 4 | 5 | N/A |
| 3. Do you like having an active role in your treatment? | 1 | 2 | 3 | 4 | 5 |  |

Please provide a comment to explain your responses:

_______________________________________________________________________________________________________________________________________________________________________________________________________________________________________________________________________________________________________________________________

The following questions relate to your experience of **exhale breath-hold**:

|  | **Not at all** | **Slightly** | **Neutral** | **Moderately** | **Very** |
| --- | --- | --- | --- | --- | --- |
| 1. How comfortably could you hold your breath in **exhale breath-hold**? | 1 | 2 | 3 | 4 | 5 |
| 2. Did you feel anxious while holding your breath in **exhale breath-hold**? | 1 | 2 | 3 | 4 | 5 |
| 3. Did you feel you could easily recover after holding your breath in **exhale breath-hold?** | 1 | 2 | 3 | 4 | 5 |

Please provide a comment to explain your responses:

_______________________________________________________________________________________________________________________________________________________________________________________________________________________________________________________________________________________________________________________________

The following questions relate to your experience of **inhale breath-hold**:

|  | **Not at all** | **Slightly** | **Neutral** | | **Moderately** | **Very** |
| --- | --- | --- | --- | --- | --- | --- |
| 1. How comfortably could you hold your breath in **inhale breath-hold**? | 1 | 2 | | 3 | 4 | 5 |
| 2. Did you feel anxious while holding your breath in **inhale breath-hold**? | 1 | 2 | | 3 | 4 | 5 |
| 3. Did you feel you could easily recover after holding your breath in **inhale breath-hold?** | 1 | 2 | | 3 | 4 | 5 |

Please provide a comment to explain your responses:

_______________________________________________________________________________________________________________________________________________________________________________________________________________________________________________________________________________________________________________________________

The following questions relate to your experience of **deep-inhale breath-hold**:

|  | **Not at all** | **Slightly** | **Neutral** | | **Moderately** | **Very** |
| --- | --- | --- | --- | --- | --- | --- |
| 1. How comfortably could you hold your breath in **deep-inhale breath-hold**? | 1 | 2 | | 3 | 4 | 5 |
| 2. Did you feel anxious while holding your breath in **deep-inhale breath-hold**? | 1 | 2 | | 3 | 4 | 5 |
| 3. Did you feel you could easily recover after holding your breath in **deep-inhale breath-hold?** | 1 | 2 | | 3 | 4 | 5 |

Please provide a comment to explain your responses:

_______________________________________________________________________________________________________________________________________________________________________________________________________________________________________________________________________________________________________________________________

Please rank the three breath-hold methods (exhale, inhale or deep-inhale breath-hold) in order of your preference, with 1 being your most preferred and 3 being your least preferred:

| **Preference** | **Breath-Hold Method** |
| --- | --- |
| 1 |  |
| 2 |  |
| 3 |  |

Please provide a comment to describe your ranking of the three methods:

_______________________________________________________________________________________________________________________________________________________________________________________________________________________________________________________________

Is there anything else you would like to tell us about your experience today?

_______________________________________________________________________________________________________________________________________________________________________________________________________________________________________________________________
